# PainVision® Apparatus for Assessment of Efficacy of Pulsed Radiofrequency Combined with Pharmacological Therapy in the Treatment of Postherpetic Neuralgia and Correlations with Measurements

**DOI:** 10.1155/2017/5670219

**Published:** 2017-03-05

**Authors:** Dong Wang, Kai Zhang, ShaoLong Han, LingZhi Yu

**Affiliations:** ^1^Department of Pain Management, Jinan Central Hospital Affiliated to Shandong University, Jinan, Shandong, China; ^2^Department of Anesthesia, Zhangqiu District Chinese Medical Hospital, Jinan, Shandong, China; ^3^Department of Neurosurgery, The Sixth People's Hospital of Jinan, Shandong, China

## Abstract

*Objective*. PainVision device was a developed application for the evaluation of pain intensity. The objective was to assess the efficacy and safety of pulsed radiofrequency (PRF) combined with pharmacological therapy in the treatment of postherpetic neuralgia (PHN). We also discussed the correlation of the measurements.* Method*. Forty patients with PHN were randomized for treatment with PRF combined with pharmacological therapy (PRF group, *n* = 20) or pharmacological therapy (control group, *n* = 20) at postoperative 48 hours. The efficacy measure was pain degree (PD) that was assessed by PainVision and visual analog scale (VAS), short form Mcgill pain questionnaire (SF-Mcgill), and numeric rate scale sleep interference score (NRSSIS). Correlations between PD, VAS, SF-Mcgill, and NRSSIS were determined.* Results*. The PD for persistent pain (PP) and breakthrough pain (BTP) at postoperative 48 hours assessed by PainVision were significantly lower in PRF group than in control group (PD-PP, *P* < 0.01; PD-BTP, *P* < 0.01). PD and VAS were highly correlated for both persistent pain (*r* = 0.453, *ρ* = 0.008) and breakthrough pain (*r* = 0.64, *ρ* = 0.001).* Conclusion*. PRF was well tolerated and superior to isolated pharmacological therapy in the treatment of PHN. PainVision device showed great value in the evaluation of pain intensity and PD had an excellent correlation with VAS and SF-Mcgill.

## 1. Introduction

Patients with postherpetic neuralgia (PHN) always suffer persistent and severe breakthrough pain (BTP) which may arise from nerve changes virus affection or immune response. BTP is characterized by brief duration (median 30 min), a severe intensity, rapid onset (less than 3 minutes), and daily frequency (more than 4 episodes per day) [1]. The evaluation of pain intensity of PHN is not frequently adequate because the patients cannot express it accurately and meticulously communicate with clinical workers. Postherpetic neuralgia (PHN) results from injury to the nerves system caused by varicella zoster virus during shingles infection [2]. Postherpetic neuralgia is one of the highest incidences of neuropathic pain (NP) syndrome and characterized by pain. The patients who recover from herpes zoster rash may be seized with months and years of pain. Varicella zoster virus is the main cause of herpes zoster (HZ). The Department of Veterans Affairs (VA) Cooperative Studies Program (CSP) 403, Shingles Prevention Study (SPS), demonstrated that live attenuated Oka/Merck VZV vaccine (zoster vaccine) reduced the HZ burden of illness (BOI) (a severity-by-duration measure of HZ pain and discomfort) by 61.1%, incidence of PHN by 66.5%, and incidence of HZ by 51.3% [3]. When varicella zoster virus (VZV) infects the body, exanthematous rash occurred at the initial stage. When VZV infects the nerves, it may just be an unbearable pain.

The incidence and severity of HZ increase with age; this is largely due to the decline of cell-mediated immunity with age [4]. The advancing of diseases depended on age and immunity of organism. PHN is more likely to occur in low immunity and elderly patients. Patients who suffered from PHN are troubled by persistent pain or paroxysmal severe pain. The pain is characterized by a variety of forms of expression because PHN impairs all the three kinds of sensory fiber, such as C, A*δ*, and A*β* fibers.

The evaluation of pain related to PHN, with visual analog scale (VAS), the short form Mcgill pain questionnaire (SF-Mcgill), and numeric rating scale sleep interference score (NRSSIS), is widely used but not considered to be perfect and accurate. In recent years, the PainVision system (PainVision PS-2100, Osachi Corporation, Japan), a device capable of quantitative assessment by substituting pain with different sensory stimulation, has been developed and used mainly in the field of anesthesiology and in pain clinics in Japan [5]. In this paper, we present a measuring device and evaluate the pain approach to the PHN. The device we applied is referred to as “PainVision PS-2100.” The using of PainVision PS-2100 makes it possible to monitor quantitative current perception threshold (CPT) and pain equivalent current (PEC). The numerical values represent the changes in peripheral sensory nerves. The experiment made by the researching is aimed at the results that the evaluation of comprehensive treatment of PHN was compared with the questionnaire and PainVision PS-2100.

## 2. Materials and Method

### 2.1. Patients

The study was approved by Jinan Central Hospital affiliated to Shandong University Institutional Review Board (IRB). The patients were informed of the condition and agreed to the treatment plan. The patients were assigned into PRF or control group randomly. We used a sealed envelope method for randomization and conducted the single-blinded study. In the study, the patients took screening procedures and determined inclusion or not. We performed the study from December 2015 to October 2016. 40 patients with PHN with abdominal and thoracic back pain were admitted to Jinan Central Hospital affiliated to Shandong University and were included in the study. All the patients have suffered the severe pain for more than 3 months.

The inclusion criteria was as follows:

1. Patients had PHN.
2. Patients had no serious diabetes and diabetic neuropathy.
3. Patients had no limb sensory and motor disorders caused by central nervous system disease.
4. Patients could communicate with the language and follow the instructions.
5. Patients accepted radiofrequency and nerve block therapy when it was necessary.

We performed a power calculation to determine the number of subjects by using power and sample size calculation system (PS: version 3.1.2, 2014). When power for uncorrected chi-squared test was over 80%, there was not a statistical difference between treatments.

### 2.2. Evaluation of Method

#### 2.2.1. Visual Analog Scale

Visual analog scale (VAS) was a 100-millimeter (mm) horizontal line labeled “no pain” at one end and “worst pain imaginable” on the other end. The patients were asked to mark on this line where the intensity of the pain existed. The distance from “no pain” to the patients' mark numerically quantifies the pain. The VAS was a simple and efficient method that correlates well with other reliable methods.

#### 2.2.2. Short Form Mcgill Pain Questionnaire (SF-Mcgill)

This questionnaire has 3 parts: the first assesses pain quality and yields a sensory score (sum of 11 adjectives: throbbing, shooting, stabbing, sharp, cramping, gnawing, hot-burning, aching, heavy, tender, and splitting, each rated on an intensity scale with 0 = none, 1 = mild, 2 = moderate, and 3 = severe), an affective score (sum of four adjectives, tiring-exhausting, sickening, fearful, and punishing-cruel related on the same intensity scale), and a total score (sum of the sensory and effective scores). The second part of the SF-MPQ consists of a 100 mm VAS of pain intensity that patients used to rate their pain during the preceding week. The third part of the SF-MPQ is a measure of present pain intensity (PPI) using a six-point scale (0 = none, 1 = mild, 2 = discomfort, 3 = distressing, 4 = horrible, and 5 = excruciating).

#### 2.2.3. Numeric Rating Scale Sleep Interference Score (NRSSIS)

The evaluation of quantity of sleep was measured by numeric rating scale (NRS) sleep interference score. It is an 11-point numerical rating scale (0 = did not interfere with sleep, 10 = completely interfere with sleep). It was included in the daily diary which was completed by the patients each day after awakening.

#### 2.2.4. PainVision Apparatus (PainVision PS-2100)

The electrode was attached to the left middle forearm of ulnar side, 1 centimeter (cm). The electrode could transmit electric current. PainVision generated the current that was pulsed for 0.3 milliseconds (ms) pulse width and 50 hertz (HZ) pulse frequency. The apparatus generated the current stimulation that compared with pain stimulation. Current perception threshold (CPT), also known as minimum perceived current, was defined as the current that had just been felt by the patients. PEC was defined as the increasing current that had been felt with the same pain intensity as the affected area by the patients. CPT and PEC were both measured three times and the mean value was recorded for calculation. We could calculate persistent pain intensity by using CPT and PEC through the following formulas:

(1) Pain  Degree=PEC−CPTCPT×100Pain  Ratio=PECCPT.

We also could evaluate breakthrough pain intensity by PainVision. Pain degree for breakthrough pain was set to CPT multiplied by a value. As the current was increasing by constant time varying rate, the patients pressed the button when they felt the intensity is the same as the moment of affected pain.

### 2.3. Therapeutic Method

All the patients are divided into 2 groups. Those in control group only accepted pharmacological treatment for ten days. The patients in PRF group accepted pharmacological treatment and minimally invasive surgery in the 8th day to treat PHN. Induction was achieved with diclofenac 75 milligrams (mg)/day, pregabalin 300 mg/day, cobamamide 1 mg/day. The minimally invasive surgery was maintained with pulsed radiofrequency (PRF) therapy, nerve block, and block of local nerve receptor. The minimally invasive surgeries were performed by the same chief physician under ultrasonographic guidance. Induction of the surgery was achieved with ropivacaine 80 mg, betamethasone compound 1 milliliter (mL), cobamamide 2 mg, lidocaine 300 mg, and 0.9% physiological saline 60 mL. In the study, the therapeutic region was first determined by the thoracic segment affected by herpes zoster, which is usually accompanied with specific neuropathic pain (NP); the lesion of one segment of dorsal rot ganglion (DRG) leads to the alternation of the nearby DRG [6]. The physician performed the puncture course under ultrasonographic (US) guidance. The needle position inside the nerve was confirmed by US (Docking Cart NZCAT, GE Medical Systems Co. Ltd, Wuxi, China) guidance. We decided to use both the motor nerve function test and the sensory nerve function test. Then the operator turned the PRF (radiofrequency generator G4, Cosman Medical Inc, Burlington, USA) into working mode to begin the treatment. The function of parameters was set to 42°C, 120 seconds, and the twice treatment for a group. 5 mL combination of liquid medicine was injected at each thoracic segment after the treatment of PRF. The needle of the length of 0.5 cm was punctured into the subcutaneous issue of the therapeutic region and liquid medicine to finish the block of local nerve receptor.

The pain intensity of PHN was assessed using the VAS and SF-Mcgill on admission and at 48 hours after surgery. The sleep interference was estimated at the same time point.

The PainVision performed the assessment of pain intensity on admission and at 48 hours after surgery. The patients were lying on the bed and the procedure was performed in a quiet room. At first, the electrode was patched onto the left forearm. The current perception threshold which indicated the feeling of stimulus was measured three times and the mean value was calculated. The CPT of left forearm was used to calculate pain degree. The CPT of other parts of the body was also assessed, such as the right forearm and the anterior and posterior parts of both ankles. Second, PET which indicated a gradually increasing current equal to the painful area was measured three times and the mean value was recorded. Pain degree for persistent pain could be got through the calculation of CPT and PEC. Third, PainVision offered 2 modes of stimulus action. One of the types was used for the evaluating of CPT and persistent pain. The current stimulus was increased constantly and steadily to a maximum of 256 uA in the procedure of assessment of persistent pain (Picture  1 in Supplementary Material available online at https://doi.org/10.1155/2017/5670219). As far as the breakthrough pain is concerned, the testing current stimulus intensity was increased sharply to the setting of whole multiple of CPT (Picture  2). Breakthrough pain could be assessed by PainVision. The compatible pain current was provided as follows: instant current was offered to the peptidases. And the compatible pain was set as a whole multiple of CPT.

Statistical analyses of the results were performed by using SPSS 15.0. The data were summarized by using the mean ± standard deviation. Treatment effects were performed with *t*-test between PRF group and control group. Comparison of the patients' basic situation between the 2 groups is performed with the chi-square analysis. The relationship between the PainVision and VAS, SF-Mcgill, and NRSSIS was statistically analyzed using Pearson's correlation coefficient. The associated factors were analyzed in PRF group. Treatment effects were reported at endpoint together with the corresponding 95% CI and *P* values. The level of significance located at *P* value of 0.05. When *P* value < 0.05, the result was considered statistically significant. The level of correlation was expressed at *ρ* value. When *ρ* value < 0.05, the result was considered to be with high degree of correlation.

## 3. Results

**Figure 1:**
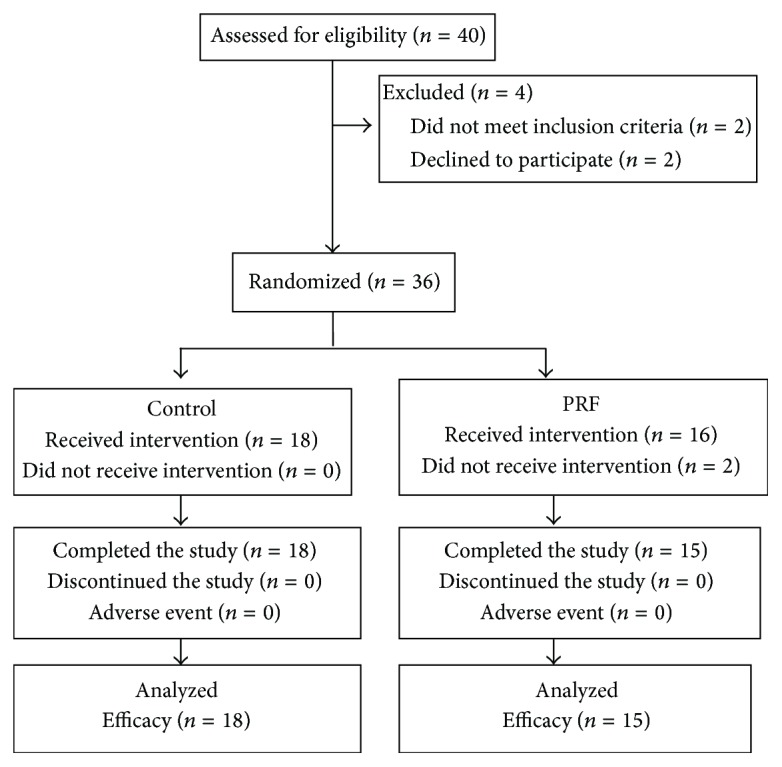
Subject flow through the trial.

A total of 40 patients (PRF, *n* = 20; control group *n* = 20) (Figure 1) were randomly with the groups balanced for baseline gender, age, weight, body mass index (BMI), height, history of diabetes, history of hypertension, duration of PHN, and affected region of the body (Table 1). We performed a power calculation and got the result power = 0.831. It was determined that there was no statistical difference between the two groups. No significant differences between PRF and control group treatment groups were identified at baseline assessments with respect to CPT, PET, PR, PD for persistent pain, PD for breakthrough pain, VAS for persistent pain, VAS for breakthrough pain, sensory score of SF-Mcgill, affective score of SF-Mcgill, total score of SF-Mcgill, visual analog scale of SF-Mcgill, present pain intensity of SF-Mcgill, and numeric rate scale sleep interference score (Table 2).

Overall 33 patients (82.5%) completed the 10-day study. Of all the patients, 18 patients accepted pharmacological therapy; 15 patients received PRF and pharmacological therapy (Figure 1).

### 3.1. Efficacy

Pain reduction at the 10-day endpoint was significantly different in control and PRF groups. Pain degree scores for both persistent and breakthrough pain at 10-day endpoint (postoperative 48 h) for pulsed radiofrequency (PRF) showed decrease and were significantly lower in the PRF group than in the control group. There was significant difference in pain ratio between the 2 groups. No differences in CPT and PEC values were detected at endpoint between the 2 groups (CPT, *P* = 0.244; PEC, *P* = 0.144). The mean of VAS for both breakthrough pain and persistent pain at on postoperative 48 h was significantly lower in the PRF group than in the control group (Table 3). Significant differences in five different parts of SF-Mcgill were detected at postoperative 48 h between the 2 groups, such as sensory score, effective score, total score, visual analog scale, and present pain scale (*P* < 0.01). At study endpoint, the PRF group and control group showed great differences in the NRS sleep interference score (*P* < 0.01) (Table 3).

**Figure 2:**
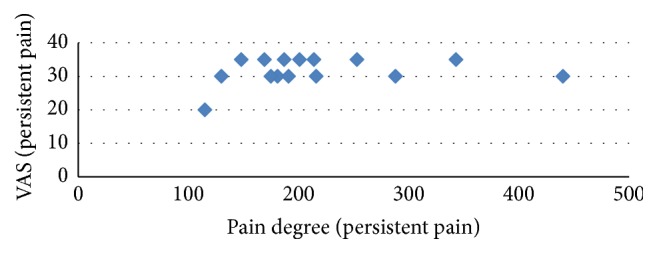
Correlation between VAS and pain degree for persistent pain at postoperative 48 hours. VAS: visual analog scale.

**Figure 3:**
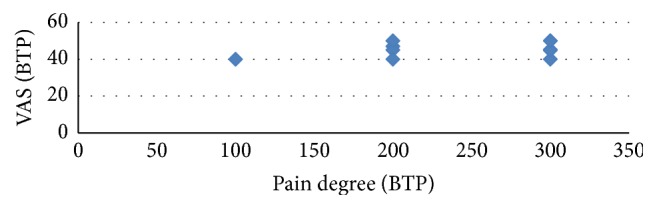
Correlation between VAS and pain degree for breakthrough pain at postoperative 48 hours. VAS: visual analog scale; BTP: breakthrough pain.

The pain degree and VAS for persistent pain at 48 hours were highly correlated in both groups (Pearson's correlation coefficient *r* = 0.453, *ρ* = 0.008) (Figure 2). The pain degree and VAS for breakthrough pain at postoperative 48 hours highly correlated in both groups (Pearson's correlation coefficient *r* = 0.64, *ρ* = 0.001) (Figure 3). The relations of pain degree for persistent pain and sensory score, effective score, total score, and present pain intensity of SF-Mcgill were highly correlated (Pearson's correlation coefficient *r* = 0.459, *ρ* = 0.007; *r* = 0.454, *ρ* = 0.008; *r* = 0.583, *ρ* = 0.001; *r* = 0.498, *ρ* = 0.003) (Table 4) in the PRF group. The relations of pain degree for breakthrough pain at endpoint (postoperative 48 hours) and VAS and SF-Mcgill that consists of five different parts were highly correlated (Pearson's correlation coefficient, VAS, *r* = 0.64, *ρ* = 0.001; SF-Mcgill, *r* = 0.548, *ρ* = 0.001; *r* = 0.323, *ρ* = 0.067; *r* = 0.526, *ρ* = 0.002; *r* = 0.522, *ρ* = 0.002; *r* = 0.0743, *ρ* = 0.001) (Table 5), except the effective score of SF-Mcgill. The pain degree for persistent pain and NRS sleep interference scale lowly correlated in the PRF group (Pearson's correlation coefficient, persistent pain, *r* = 0.276, *ρ* = 0.12; breakthrough pain, *r* = 0.31, *ρ* = 0.079).

No treatment-related serious adverse events occurred, such as pneumothorax, nerve injury, and infection, hemorrhage, and haematoma.

## 4. Discussion

PainVision took the advantage of the assessment of pain intensity, including persistent pain and breakthrough pain. We found that a majority of PHN treatment via PRF had successful pain relief with emotional improvement.

PHN is related to several mechanisms. The potential factors are associated with age and duration of disease. Herpes zoster and its complications, especially postherpetic neuralgia, are associated with substantial morbidity among older adults [7]. In patients with HZ, PHN is a common complication and affects the quality of the patients. It is usually defined as pain persisting for at least 3 months after the rash onset [8]. All patients were 50 years or older and had a duration of more than 3 months. PHN frequently brings out severe pain, and the underlying mechanism remains unknown.

The virus causes severe sensory nerve diseases manifestations. Some populations include nervous system damage (degeneration of afferent fibers) leading to a sensitized hyperexcitability pain state, second-order neurons becoming responsive to A fibers originating from low-threshold mechanoreceptors, and central nervous involvement in the form of loss of inhibitory interneurons [9]. PHN invades all sensory fibers, such as C fibers, A*β*fibers, and A*δ* fibers, and cause sharp pain, burning pain, allodynia, or hypersensitivity [6].

As far as the patients with PHN are concerned, there are a lot of differences in nature and duration of pain. As is the case for other neuropathic pain conditions, it is characterized by the combination of a relative small number of positive (continuous pain such as burning and paroxysmal pain, brush-evoked allodynia) and negative (hypoesthesia) symptoms that probably reflect distinct pathophysiological mechanisms [10]. BTP has been reported to be a relevant problem in patients with PHN, such as physical, psychological, and economic burden. It is defined as serious pain and horrible pressure that should be treated timely and effectively.

In the study, patients in PRF group experienced substantial improvement in mood, relief of pain intensity of persistent pain, and BTP. Currently, the measures have been widely used in clinic work, such as VAS, SF-Mcgill, and NRSSIS. Of patients with neuropathic pain who take prescription medications for such pain, more than one-quarter also take medications for anxiety, depression, or sleep [11]. In addition, the patients with PHN are also troubled with sleep interference because of chronic pain. On the one hand, when the patients are troubled by pain, their sleep is disturbed. On the other hand, the poor sleep pushes on the degree of pain.

Compared with VAS, SF-Mcgill, and NRS sleep interference score, the pain degree which was obtained by using PainVision device showed great value in assessing pain intensity. PainVision was a quantitative analysis device and could be used for the evaluation of both perception and pain intensity. Currently, it has been widely used in the department of anesthesiology and pain management. The device may be used to objectively evaluate the function of nerve, chronic pain, and acute pain. It took the advantage of being noninvasive. The transmission of nerve depends on sensory nerves that are divided into unmyelinated nerve and myelinated nerve. Sensory nerve was composed of 3 types of nerves, including unmyelinated C fibers, myelinated A*β* fibers, and A*β* fibers. The nerves take different information conduction. C fibers transmitted sensory information of nonpainful stimuli. According to A fibers, the information of painful stimuli is conveyed by them.

PainVision stimulates A*β* fibers and A*δ* fibers. PainVision device could generate the current of 0.3 ms width and 50 HZ frequency that was equivalent to the range of 50 Hz to 2000 Hz stimulation. The nerve fibers contain 3 different types, such as A*β* fibers, A*δ* fibers, and C fibers, respectively, corresponding to 2000 Hz stimulation, 250 Hz stimulation, and 5 Hz stimulation. The PainVision simulation generated action potentials in myelinated A*β* fibers, whereas action potentials were generated in myelinated A*δ* fibers. It could not stimulate unmyelinated C fibers. By stimulating A*β* and A*δ* fibers, CPT and PEC are measured by the PainVision device. Then the degree of pain is calculated from the two values. The stimuli of PainVision could cause shooting pain, sharp pain, hot-burning pain, and allodynia that are similar to the way of PHN. PainVision device contained 3 types of measuring procedure, such as CPT measurement, pain measurement, and pain level measurement, respectively, corresponding to the measurement of current perception threshold, the assessment of persistent pain, and the assessment of BTP. Because individual pain threshold is evaluated first for accurate subsequent measurement with the device, pain intensity can be quantitatively compared among patients [12].

PainVision could have significant effects on evaluation of pain intensity, including both PP and BTP. It takes the advantage of the quantitative comparison of pain degree. Although PainVision only stimulates A*β* and A*δ* fibers, the stimulation can be equivalent to pain. The stimulus of PainVision was similar to the impact of the pain. At first, it generates painless sensory stimulus where the patients feel a slight sensation and gets CPT. In a subsequent test, it gives more and generated pain sensory stimulation that is equivalent to the suffered pain and PET is received. PD is calculated from the formula with CPT and PEC. The degree of pain can be quantitatively assessed for the patients. When the persistent pain is measured, the current stimulus increases gradually to match the pain level. As far as the breakthrough pain is concerned, the stimulus rose steeply from the lower level to the set value. When the stimulus acts on the body, the feelings are similar to acupuncture, numbness, and power and the similarity made the measurement more accurate. The reduction in the average of PD was always associated with significant reduction in VAS, SF-Mcgill, and NRSSIS at PRF group. Correlation analysis used in the study described the relationship and influence between variables under PRF. The high correlation indicates that the device was as efficient as VAS and SF-Mcgill. The effective score of SF-Mcgill was not highly related with PD of BTP. The relationship between BTP and emotion remains to be studied. The sleep was improved, but the correlation was low between PD and NRSSIS.

In the process, a normal CPT was defined as the range from the mean −1 SD to +1 SD using the CPT values from the healthy subjects [13]. When CPT is out of range and below it, the situation is diagnosed as hyperaesthesia. On the contrary, it is considered hypoesthesia. PainVision can be used to assess the function of peripheral nerve. PET also is calculated by the way. CPT can be influenced by many factors, such as age and diseases of peripheral nerve. For example, paresthesia of the infraorbital nerve region was caused by the damaged A*δ* and C fivers [14]. We found that although some diabetic patients had higher CPT values than control subjects, others had lower CPT values [15]. The calculation of PD takes into account that CPT may be affected by situation. PD is expressed by the ratio of the difference of PEC and CPT. The difference between PEC and CPT is defined as the part of value used to generate pain. The ration represents the fact that the current of generating pain is a multiple of the current of sensory part. The method can exclude all kinds of influence of CPT. Another calculation is pain ratio. It is expressed by the ratio of PEC and CPT. It can be considered that PEC is a multiple of sensory part. Because PEC is composed of the stimuli of generating pain and sensory, the pain ratio does not take advantage of PD.

Patients with PHN who experience a variety of different pains may have different effects on the pain and on how it affects quality of life. Analgesic, cardiovascular, gastrointestinal, endocrine, and central nervous system drugs constitute the most prevalent therapeutic classes [16]. Drugs are commonly used to treat PHN, including gabapentin and pregabalin, and opioids. Peripheral nerve electricity modulation can reduce allodynia for a long time [17]. Pulsed radiofrequency (PRF) may act on some kind of nerves. Compared with the RF thermal ablation, ultrasound guided PRF is safe, applicable, and effective for PHN. Compared to the temperature radiofrequency, PRF takes more advantages. The results of previous experiments collectively suggested that PRF appeared to provide neuromodulation in response to painful stimuli without changing the morphology of motor and sensitive fibers; it probably works with a temperature independent pathway mediated by changing electric fields [18]. Therefore, in pain management department, pharmacological therapy and PRF are performed during the therapeutic process. The results of the study indicated that the combined treatment methods could significantly reduce the pain intensity of persistent pain and breakthrough pain and improve the patients' mood and sleep quality.

There are both limitations and advantages in our study. As far as the limitations are concerned, the number of the patients is not big enough and the number of samples is more persuasive. The inclusive institution is one institution in China and the study may be probably restricted by regional limitation. However, one of the advantages is that the combination therapy was performed showing its value in clinic work. Second, the patients did use strong opioid analgesics and reduced the addiction and dependence. Third, the pain intensity was detailed qualification by PainVision. Fourth, the persistent pain and momentary pain were assessed, respectively, and made more accurate assessment of the patients' condition. The further study for other diseases and treatments needs to be performed.

## 5. Conclusion

PRF was well tolerated and superior to isolated pharmacological therapy in the treatment of PHN. PainVision device could show great value in the evaluation of pain intensity of PHN and PD showed an excellent correlation with VAS and SF-Mcgill.

## Supplementary Material

1.Picture 1 Photo of PainVision and the working procedure. The electrode was attached to the left middle forearm of ulnar side 1 centimeter (cm). Patientsgrasp a switch with right hand. 2.Picture It is the screen of PainVision when the measurement of breakthrough pain (BTP) is working. 3.Picture 3.It is the screen of PainVision when the measurement of persistent pain (PP)or current perception threshold (CPT) is working. 4. Picture 4. the procedure of the PRF surgery under ultrasonographic (US) guidance.

## Figures and Tables

**Table 1:** Demographic baseline characteristics.

Characteristics	Control (*n* = 18)	PRF (*n* = 15)	*P* value
Ration (%)	55	45	
Mean age, year(SD)	72.22 ± 10.41	67.73 ± 13.94	0.161
Sex: female (%)	13 (72)	10 (66.7)	0.082
Weight (kg)	66.72 ± 11.24	63.03 ± 9.28	0.482
BMI (kg/m^2^)	25.73 ± 4.75	23.05 ± 3.11	0.404
Height (cm)	161.44 ± 9.08	166.00 ± 8.90	0.878
Diabetes (%)	3 (16)	2 (13)	0.141
Hypertension (%)	5 (27)	6 (40)	0.105
Duration (week)	26 ± 7	26 ± 6	0.477
Alcohol user (%)	4 (22)	4 (27)	0.62
Nicotian (%)	5 (27)	5 (33)	0.551

BMI: body mass index, SD: standard deviation, and PRF: pulsed radiofrequency.

**Table 2:** Basic efficacy outcomes of patient on admission.

	Control group (*n* = 18)	PRF group (*n* = 15)	Difference (SE)	95% CI	*P*
CPT (uA)	13.68 ± 6.12	17.21 ± 9.43	−3.52 (2.72)	(−9.07,2.03)	0.205
PEC (uA)	66.61 ± 18.12	81.55 ± 42.37	−14.95 (11.01)	(−37.4,7.5)	0.184
PR	5.36 ± 1.53	5.32 ± 2.19	0.03 (0.65)	(−1.29,1.36)	0.96
PD-PP	435.56 ± 152.93	431.93 ± 219.1	3.62 (64.93)	(−128.8,136.1)	0.956
VAS-PP (mm)	60.83 ± 7.91	60.00 ± 8.24	0.83 (2.82)	(−4.91,6.58)	0.769
PD-BTP	427.78 ± 46.09	426.67 ± 45.74	0.01 (0.16)	(−0.32,0.34)	0.945
VAS-BTP (mm)	76.11 ± 5.57	76.40 ± 5.49	−0.29 (1.93)	(−4.23,3.66)	0.882
SF-Mcgill					
Sensory score	5.61 ± 0.61	5.20 ± 0.77	0.41 (0.24)	(−0.80,0.90)	0.097
Effective score	3.60 ± 1.10	3.60 ± 0.91	−0.44 (0.35)	(−0.77,0.68)	0.901
Total score	9.22 ± 1.14	8.80 ± 1.37	0.42 (0.43)	(−0.46,1.30)	0.337
VAS (mm)	60.83 ± 7.91	60.00 ± 8.24	0.83 (2.82)	(−4.91,6.58)	0.769
PPI	4.00 ± 0.59	3.87 ± 0.35	−0.13 (0.17)	(−0.22,0.49)	0.451
NRSSIS	5.11 ± 0.58	3.80 ± 0.56	1.31 (0.20)	(0.90,1.72)	0.101

PRF: pulsed radiofrequency; CPT: current perception threshold; PEC: pain equivalent current; PR: pain ratio; PD: pain degree; PP: persistent pain; VAS: visual analog scale; BTP: breakthrough pain; SF-Mcgill: short form Mcgill pain questionnaire; PPI: present pain intensity; NRSSIS: numeric rate scale sleep interference score; SE: standard error; CI: confidence interval.

**Table 3:** Efficacy outcomes of patient at postoperative 48 hours (10 days from admission).

	Control group (*n* = 18)	PRF group (*n* = 15)	Difference (SE)	95% CI	*P*
CPT (uA)	13.71 ± 3.95	15.46 ± 4.52	−1.75 (1.47)	(−4.75,1.26)	0.244
PEC (uA)	54.73 ± 13.14	47.62 ± 14.05	7.10 (4.74)	(−2.56,16.77)	0.144
PR	4.07 ± 1.07	3.09 ± 0.83	0.97 (0.34)	(0.28,1.66)	0.007
PD-PP	319.83 ± 106.72	216.73 ± 85.31	103.1 (34.13)	(33.45,172.7)	0.005
VAS-PP (mm)	43.06 ± 8.25	31.67 ± 4.08	11.39 (2.34)	(6.61,16.16)	0.001
PD-BTP	377.78 ± 73.20	260.00 ± 63.24	117.78 (24.08)	(68.66,166.9)	0.001
VAS-BTP (mm)	56.11 ± 8.95	45.13 ± 3.31	10.98 (2.34)	(6.28,15.73)	0.001
SF-Mcgill					
Sensory score	4.44 ± 0.62	3.20 ± 0.41	1.24 (0.19)	(0.87,1.63)	0.001
Effective score	2.51 ± 0.79	1.80 ± 0.68	0.70 (0.26)	(0.17,1.23)	0.011
Total score	7.00 ± 1.08	5.00 ± 0.85	2.00 (0.35)	(1.30,2.70)	0.001
VAS (mm)	43.06 ± 8.25	31.67 ± 4.08	11.39 (2.34)	(6.61,16.16)	0.001
PPI	3.56 ± 0.70	2.53 ± 0.52	1.02 (0.21)	(0.58,1.47)	0.001
NRSSIS	3.72 ± 0.46	2.60 ± 0.51	0.68 (0.17)	(0.33,1.02)	0.001

PRF: pulsed radiofrequency; CPT: current perception threshold; PEC: pain equivalent current; PR: pain ratio; PD: pain degree; PP: persistent pain; VAS: visual analog scale; BTP: breakthrough pain; SF-Mcgill: short form Mcgill pain questionnaire; PPI: present pain intensity; NRSSIS: numeric rate scale sleep interference score; SE: standard error; CI: confidence interval.

**Table 4:** Relationship between changes for persistent pain in PD, VAS, SF-Mcgill, and NRSSIS at postoperative 48 hours.

	PD vs. VAS	SF-Mcgill					PD vs. NRSSIS
		PD vs. sensory score	PD vs. effective score	PD vs. total score	PD vs. VAS	PD vs. PPI	
*γ*	0.453	0.459	0.458	0.583	0.453	0.498	0.276
*ρ*	0.008	0.007	0.008	0.001	0.008	0.003	0.12
*n*	33	33	33	33	33	33	33

PD: pain degree, VAS: visual analog scale, SF-Mcgill: short form Mcgill pain questionnaire, NRSSIS: numeric rate scale sleep interference score, PPI: present pain intensity, and vs.: versus.

**Table 5:** Relationship between changes for breakthrough pain in PD, VAS, SF-Mcgill, and NRSSIS at postoperative 48 hours.

	PD vs. VAS-BTP	SF-Mcgill					PD vs. NRSSIS
		PD vs. sensory score	PD vs. effective score	PD vs. total score	PD vs. VAS	PD vs. PPI	
*γ*	0.64	0.548	0.323	0.526	0.522	0.0743	0.31
*ρ*	0.001	0.001	0.067	0.002	0.002	0.001	0.079
*n*	33	33	33	33	33	33	33

PD: pain degree, VAS: visual analog scale, SF-Mcgill: short form Mcgill pain questionnaire, NRSSIS: numeric rate scale sleep interference score, PPI: present pain intensity, vs.: versus, and VAS-EP: visual analog scale for breakthrough pain.
