# Supplementary figures and images for: PainVision® Apparatus for Assessment of Efficacy of Pulsed Radiofrequency Combined with Pharmacological Therapy in the Treatment of Postherpetic Neuralgia and Correlations with Measurements
Source: Biomed Res Int. 2017 Mar 5;2017:5670219. doi: 10.1155/2017/5670219 (PMC5357555; doi:10.1155/2017/5670219)

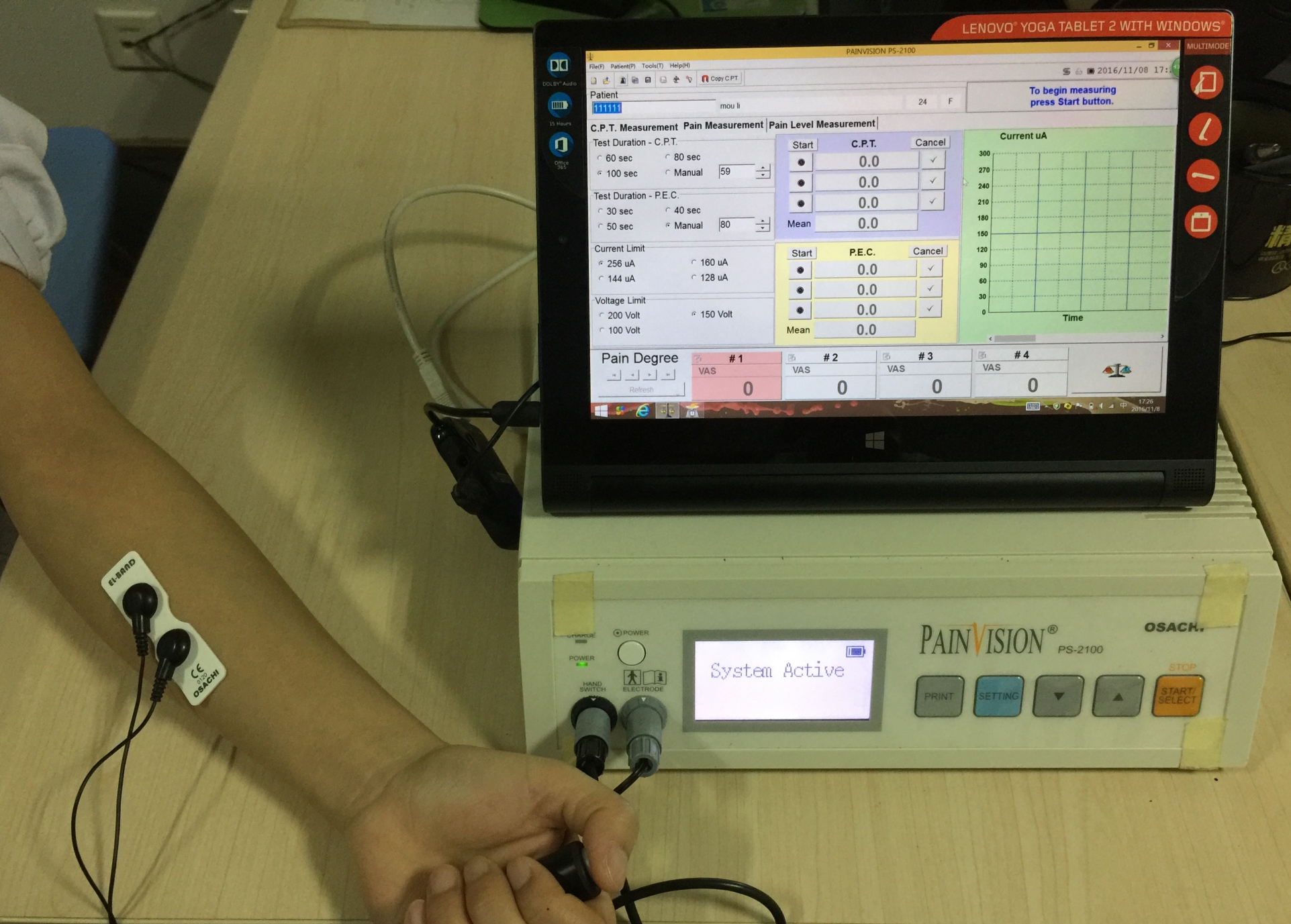

Supplement: Supplementary file 1 — 1.Picture 1 Photo of PainVision and the working procedure. The electrode was attached to the left middle forearm of ulnar side 1 centimeter (cm). Patientsgrasp a switch with right hand. 2.Picture It is the screen of PainVision when the measurement of breakthrough pain (BTP) is working. 3.Picture 3.It is the screen of PainVision when the measurement of persistent pain (PP)or current perception threshold (CPT) is working. 4. Picture 4. the procedure of the PRF surgery under ultrasonographic (US) guidance. [file 5670219.f1.zip › procedure of test.jpg]

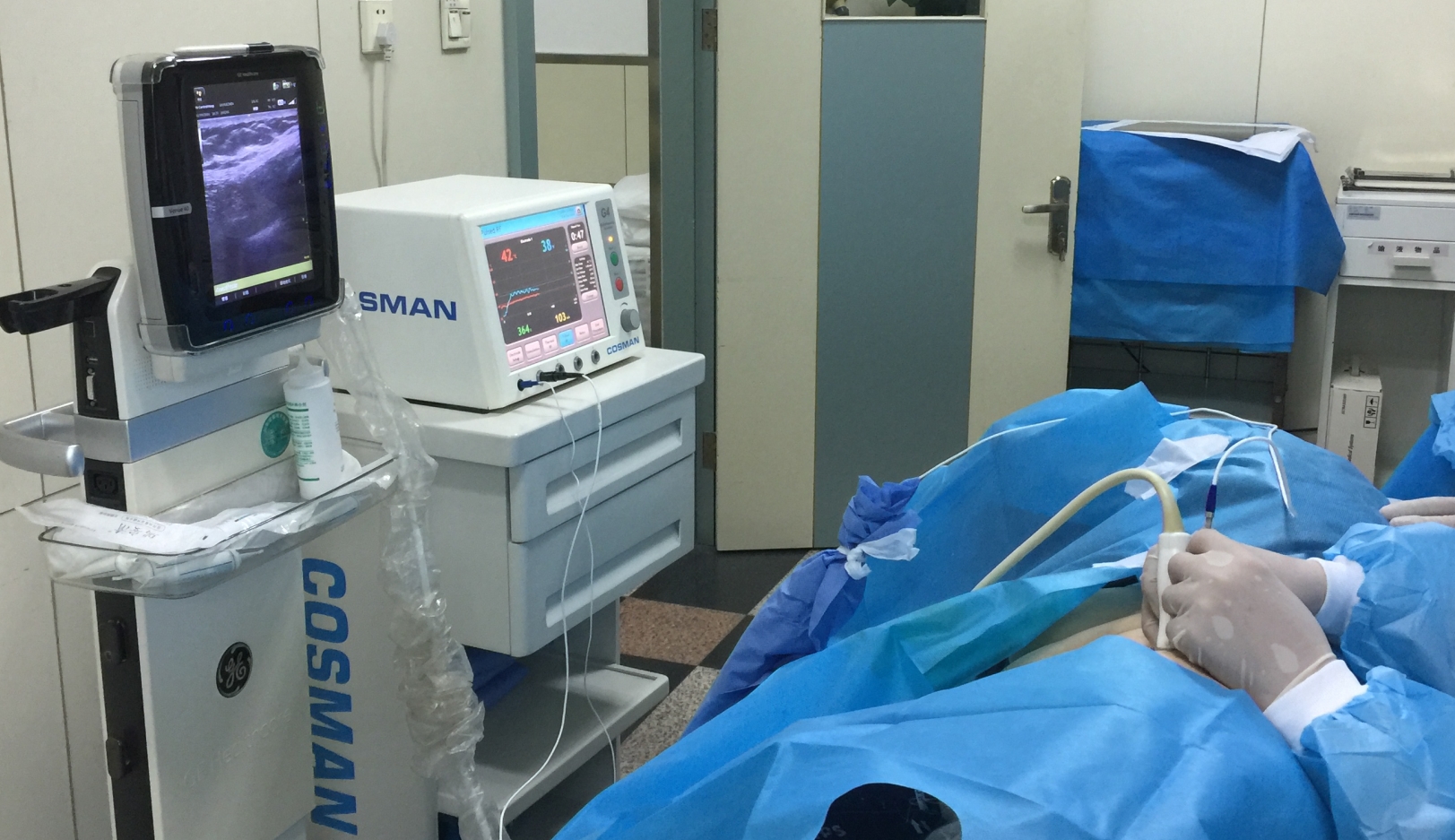

Supplement: Supplementary file 1 — 1.Picture 1 Photo of PainVision and the working procedure. The electrode was attached to the left middle forearm of ulnar side 1 centimeter (cm). Patientsgrasp a switch with right hand. 2.Picture It is the screen of PainVision when the measurement of breakthrough pain (BTP) is working. 3.Picture 3.It is the screen of PainVision when the measurement of persistent pain (PP)or current perception threshold (CPT) is working. 4. Picture 4. the procedure of the PRF surgery under ultrasonographic (US) guidance. [file 5670219.f1.zip › surgery peocedure.jpg]

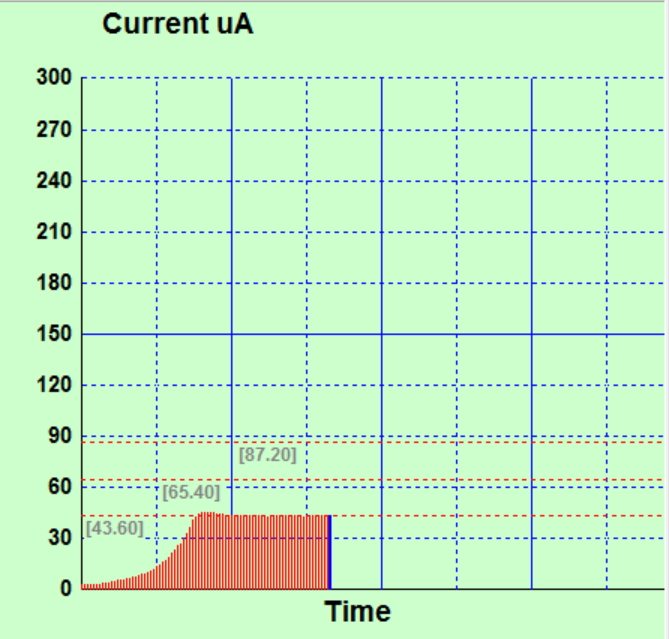

Supplement: Supplementary file 1 — 1.Picture 1 Photo of PainVision and the working procedure. The electrode was attached to the left middle forearm of ulnar side 1 centimeter (cm). Patientsgrasp a switch with right hand. 2.Picture It is the screen of PainVision when the measurement of breakthrough pain (BTP) is working. 3.Picture 3.It is the screen of PainVision when the measurement of persistent pain (PP)or current perception threshold (CPT) is working. 4. Picture 4. the procedure of the PRF surgery under ultrasonographic (US) guidance. [file 5670219.f1.zip › Test of breakthough pain.png]

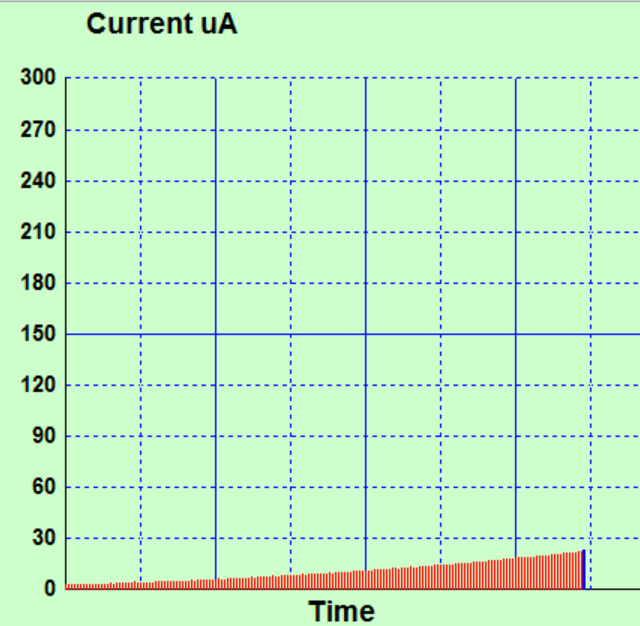

Supplement: Supplementary file 1 — 1.Picture 1 Photo of PainVision and the working procedure. The electrode was attached to the left middle forearm of ulnar side 1 centimeter (cm). Patientsgrasp a switch with right hand. 2.Picture It is the screen of PainVision when the measurement of breakthrough pain (BTP) is working. 3.Picture 3.It is the screen of PainVision when the measurement of persistent pain (PP)or current perception threshold (CPT) is working. 4. Picture 4. the procedure of the PRF surgery under ultrasonographic (US) guidance. [file 5670219.f1.zip › Test of persistent pain.png]
